# Atrial Fibrillation and Retinal Stroke

**DOI:** 10.1001/jamanetworkopen.2024.53819

**Published:** 2025-01-09

**Authors:** Jay B. Lusk, Vinit Nalawade, Lauren E. Wilson, Ailin Song, Matthew Schrag, Valerie Biousse, Oana Dumitrascu, Sven Poli, Jonathan Piccini, Bradley Hammill, Fan Li, Ying Xian, Emily O’Brien, Brian Mac Grory

**Affiliations:** 1 Department of Family Medicine, University of North Carolina, Chapel Hill; 2 Department of Neurology, Duke University School of Medicine, Durham, North Carolina; 3 Department of Population Health Sciences, Duke University School of Medicine, Durham, North Carolina; 4 Department of Ophthalmology, Duke University School of Medicine, Durham, North Carolina; 5 Department of Neurology, Vanderbilt University School of Medicine, Nashville, Tennessee; 6 Department of Ophthalmology, Emory University School of Medicine, Atlanta, Georgia; 7 Department of Neurology, Mayo Clinic Alix School of Medicine, Phoenix, Arizona; 8 Department of Neurology & Stroke, University of Tübingen, Tübingen, Germany; 9 Hertie Institute for Clinical Brain Research, University of Tübingen, Tübingen, Germany; 10 Department of Medicine, Duke University School of Medicine, Durham, North Carolina; 11 Duke Clinical Research Institute, Durham, North Carolina; 12 Department of Statistical Science, Duke University, Durham, North Carolina; 13 Department of Biostatistics and Bioinformatics, Duke University, Durham, North Carolina; 14 Department of Neurology, UT Southwestern Medical Center, Dallas, Texas; 15 Department of Medicine, UT Southwestern Medical Center, Dallas, Texas; 16 Department of Population and Data Science, UT Southwestern Medical Center, Dallas

## Abstract

**Importance:**

Atrial fibrillation (AF) is the most common, chronic, cardiac arrythmia in older US adults. It is not known whether AF is independently associated with increased risk of retinal stroke (central retinal artery occlusion), a subtype of ischemic stroke that causes severely disabling visual loss in most cases and is a harbinger of further vascular events.

**Objective:**

To determine whether there is an association between AF and retinal stroke.

**Design, Setting, and Participants:**

This retrospective cohort study was performed between July 2023 and May 2024 using computerized inpatient, outpatient, emergency department, and skilled nursing facility claims files for a 5% sample of US fee-for-service Medicare beneficiaries aged 66 years and older between 2000 and 2020. Follow-up ended at death, conclusion of fee-for-service Medicare coverage, end of the study period, or loss to follow-up of 85% of the study cohort.

**Exposure:**

AF, based on validated *International Classification of Diseases, Ninth Revision, Clinical Modification* and *International Statistical Classification of Diseases, Tenth Revision, Clinical Modification* diagnosis codes.

**Main Outcomes and Measures:**

The primary end point was incident retinal stroke in the primary diagnostic position of a single claim in any venue of care. Secondary end points included retinal stroke in any position of a single claim, 1 positive control end point (cerebral ischemic stroke), and 4 negative control end points (central retinal vein occlusion, urinary tract infection, humeral fracture, and cataract). Unadjusted and adjusted hazard ratios (HRs) and rate differences were computed across matched and overlap-weighted cohorts with and without AF (defined as 1 inpatient claim or 2 outpatient claims within a 365-day period).

**Results:**

In total, 1 090 144 patients (591 400 female [54.3%]; mean [SD] age, 76.92 [7.09] years) were included in the study; 545 072 patients had AF and 545 072 were matched controls. The median (IQR) follow-up was 45 (18 to 90) months. In total, 1333 patients with AF (rate, 0.55 per 1000 person-years) and 1082 AF-free matched controls (rate, 0.50 per 1000 person-years) experienced retinal stroke. The cause-specific, adjusted HR of retinal stroke after overlap weighting was 1.14 (95% CI, 1.02 to 1.28; adjusted rate difference, 0.05 [95% CI, −0.01 to 0.11]). AF was associated with cerebral ischemic stroke (adjusted HR, 1.73 [95% CI, 1.69 to 1.76]; adjusted rate difference, 10.11 [95% CI, 9.72 to 10.49]). Of 4 prespecified negative control end points, AF was not associated with central retinal vein occlusion but was associated with urinary tract infection, cataract, and humeral fracture.

**Conclusions and Relevance:**

In this cohort study of Medicare beneficiaries aged 66 years and older, AF was independently associated with retinal stroke. The magnitude of the association was small, and a contribution from residual, unmeasured confounding could not be excluded.

## Key Points

**Question:**

Is atrial fibrillation associated with retinal stroke?

**Findings:**

In this cohort study of 1 090 144 Medicare beneficiaries aged 66 years or older, the rate of retinal stroke was 0.55 per 1000 person-years among beneficiaries with atrial fibrillation vs 0.50 per 1000 person-years among matched beneficiaries without atrial fibrillation, a result that was statistically significant after adjustment for key covariates.

**Meaning:**

These findings suggest that among older adults enrolled in Medicare, atrial fibrillation is associated with an increased hazard rate of retinal stroke.

## Teaser

This cohort study examines whether there is an association between atrial fibrillation and retinal stroke in US adults aged 66 years and older.

## Introduction

Atrial fibrillation (AF) is the most common, chronic, cardiac arrythmia affecting older US adults^1^ and increases a person’s risk of having a stroke.^2^ Stroke in the setting of AF tends to be highly disabling because AF is associated with blockages of the brain’s largest arteries, which cause crippling deficits in strength, swallowing, speech, and sight.^3,4^ Recently, the National Heart, Lung, and Blood Institute identified an unmet need to improve detection of patients harboring undiagnosed AF including by using clinical features.^5^ There may be 700 000 such patients in the US^6^ who are at ongoing risk of stroke.

Retinal stroke (central retinal artery occlusion, colloquially known as *eye stroke*) is a rare form of ischemic stroke^7^ that, in most cases, causes severely disabling loss of sight in 1 eye.^8^ The prevailing paradigm is that retinal stroke arises because of carotid artery narrowing due to calcium or cholesterol plaque formation.^9^ However, more than one-half of people with retinal stroke do not have high-grade carotid narrowing,^10^ and 30% of people with retinal stroke do not have even mild carotid disease.^11^ It is not known whether AF is associated with the risk of retinal stroke.

Several prior conflicting observations informed the present study. First, there is a higher prevalence of AF in patients with retinal stroke than in the population at large.^12,13^ Second, we have demonstrated previously^14^ that AF-free patients with retinal stroke and with a cardiac monitoring device in situ had a higher hazard of subsequent AF detection than matched control patients and an equivalent hazard to patients with cerebral ischemic stroke. Third, a nationwide study^15^ in Taiwan described a higher incidence of retinal stroke in patients with AF than in the general population but did not account for baseline vascular risk. Fourth, we previously conducted a population-based study^16^ in the US that did not show an association between AF and retinal stroke, but that study was limited to claims from inpatient and emergency department (ED) encounters only.

Investigating an association between AF and retinal stroke may inform whether cardiac monitoring is necessary in patients with cryptogenic retinal stroke. A scientific statement from the American Heart Association and American Stroke Association identified the unmet need to improve the field’s understanding of the risk factors for retinal stroke.^8^ To address this need and to overcome the limitations of prior work, a population-based study was designed^17^ with the objective to examine this association in a 5% sample of fee-for-service Medicare beneficiaries.

## Methods

### Study Design

This study was performed in accordance with a 2-part, prespecified, published, statistical analysis plan,^17^ of which the present study is the first part. This is a retrospective cohort study that used data from a sample of 5% of fee-for-service Medicare beneficiaries from 2000 to 2020. The requirement for informed consent was waived by the institutional review board of the Duke University School of Medicine because the data are publicly available and limited, in accordance with 45 CFR §46. This study was conducted under the auspices of a data usage agreement between Duke University and the Centers for Medicare & Medicaid Services. This study is reported in accordance with the Strengthening the Reporting of Observational Studies in Epidemiology (STROBE) reporting guideline and Reporting of Studies Conducted Using Observational Routinely-Collected Data (RECORD) guidelines.^18^

### Study Setting

Data were derived from the computerized files of US Medicare, a health insurance program that provides coverage to persons aged 65 years or older or younger persons with certain chronic medical conditions, such as dialysis-dependent end-stage kidney disease. Files used for the present study included the Master Beneficiary Summary File (which includes unique identifiers for each beneficiary, enrollment status, and data on mortality), as well as claims files for office-based care, outpatient clinics, inpatient care, ED, nursing facilities, skilled nursing facilities, home care, and custodial care facilities.

### Study Population

Medicare beneficiaries aged 66 years or older with at least 12 months of continuous enrollment before their assigned study index date (defined later) were included. Patients older than 90 years at first potential index date were excluded from the study population. First, the AF cohort was derived by applying study selection criteria. Then, each beneficiary was matched on a 1:1 basis with an AF-free control patient (see the Statistical Analysis subsection). The index date was assigned as the first date of AF diagnosis (see the Exposures subsection) in the AF cohort and July 1 of a randomly selected year of study eligibility for each AF-free control subject. This approach mandates that each beneficiary could contribute only a single record to the study. Patients with any claims for AF not satisfying the study definition (see the Exposures subsection) were excluded from the study, as were patients with a claim for atrial flutter (unless they also had an AF diagnosis by the study definition). Beneficiaries with AF were not eligible for use as controls in the years before AF incidence because of a concern that subclinical AF may be present in a beneficiary for many years before the entry of an AF claim. Beneficiaries with any claim for retinal stroke (see the End Points subsection) in the 12 months before the study index date were excluded.

### Exposures

The primary exposure was AF. AF was defined via *International Classification of Diseases, Ninth Revision, Clinical Modification (ICD-9-CM)* code 427.31 or *International Statistical Classification of Diseases, Tenth Revision, Clinical Modification (ICD-10-CM)* code I48.0, I48.1, I48.2, or I48.91 in either 1 inpatient claim or 2 outpatient claims within a 365-day period, in keeping with previously published methods.^19,20,21^

### End Points

The primary end point of this study was retinal stroke identified by the previously validated^16^ *ICD-9-CM* code 362.31 (for claims before October 1, 2015) and the corresponding *ICD-10-CM* code H34.1x (for claims on or subsequent to October 1, 2015) in the primary diagnostic position within an inpatient, outpatient, ED, or skilled nursing facility claim. Secondary end points were (1) retinal stroke in any diagnostic position and (2) any form of retinal stroke (including branch retinal artery occlusion and any other diagnosed retinal artery occlusion). To assess the validity of each model, a positive control end point (cerebral ischemic stroke, defined according to previously validated methods^22^) was included. To assess for the presence of residual confounding, 4 negative control end points (central retinal vein occlusion, urinary tract infection [UTI], cataract, and humeral fracture, each according to previously published methods^23^) were included.

### Statistical Analysis

This analysis was performed between July 2023 and May 2024 using routinely collected data; however, study power was estimated before commencement of this analysis to provide context to any results obtained. A total of 570 000 patients (285 000 with AF and 285 000 without) was calculated to confer 90% power to detect a hazard ratio (HR) of 2 at an α = .05. This assumed an event rate of 22 cases per 100 000 population per year of follow-up in the AF group (an unadjusted rate of 45 cases per 100 000 population had been reported previously^15^) and an event rate of 11 cases per 100 000 population per year in the control group (consistent with a previous report^24^).

Descriptive statistics were computed, including counts and percentages for categorical variables and medians with (IQRs) for continuous variables. Between-group differences are outlined by means of absolute standardized differences. Descriptive statistics presented in the text are unweighted statistics, and weighted estimates are presented in eTables 1, 2, and 3 in Supplement 1.

To control for baseline covariate imbalance, a 2-stage approach was used: (1) a matching procedure followed by (2) propensity score–overlap weighting. First, a logistic regression model was used to derive scores reflecting the propensity to have AF vs not have AF. Variables included in the model were chosen a priori and included key demographic and clinical characteristics and the Charlson Comorbidity Index score (coded as 0, 1, 2, 3, or ≥4). All variables with claims-based definitions are outlined in a previous publication.^17^ The social construct of race was included because it is known to be associated with the risk of other forms of stroke (such as cerebral ischemic stroke) and because there are pervasive inequities in stroke care across racial groupings.^25^ Data on race and ethnicity were obtained from the Master Beneficiary Summary File. After derivation of propensity scores, each eligible patient with AF was matched on a 1:1 basis with a control patient without AF in 2 parts: (1) on an exact basis based on age and year of presentation and then (2) using propensity score matching with a caliper of 0.1. The rationale for matching on an exact basis on age and year of presentation was that a differential rate of death or secular trends in coding patterns could introduce bias to the study. Then, propensity scores were re-estimated in the matched cohorts and overlap weighting^26,27,28^ was used (a technique that weighs individual patients in accordance with their probability of being in opposite group and exhibits greater precision than other weighting methods).

A Cox model was fit and, after verifying the assumption of proportional hazards, the hazard of retinal stroke was computed in both the AF and control groups. The cause-specific adjusted HR (aHR) with its corresponding 95% CI was derived for each end point, with death as a competing risk. Unadjusted and adjusted rate differences were calculated from each model. Censoring occurred under 4 circumstances: (1) death, (2) end of fee-for-service coverage, (3) end of the study period, and (4) when 85% of the study cohort had been lost to follow-up. Censoring was assumed to be noninformative. This approach was replicated for each secondary end point, the positive control end point, and the 4 negative control end points. A falsification exposure analysis was conducted wherein acute UTI was considered the exposure and retinal stroke the end point. A sensitivity analysis was performed wherein censoring did not occur at 85% loss to follow-up of the entire study cohort. Missing data were not imputed for any study end points.

A 2-sided α = .05 was considered indicative of statistical significance for all statistical tests. Because the widths of the presented 95% CIs were not adjusted for multiplicity, all point estimates and their corresponding 95% CIs for each secondary end point should be treated as hypothesis-generating only. Analyses were performed using SAS statistical software version 9.4 (SAS Institute) and R statistical software version 4.0.2 (R Project for Statistical Computing).

## Results

### Baseline Characteristics

**Figure 1.**
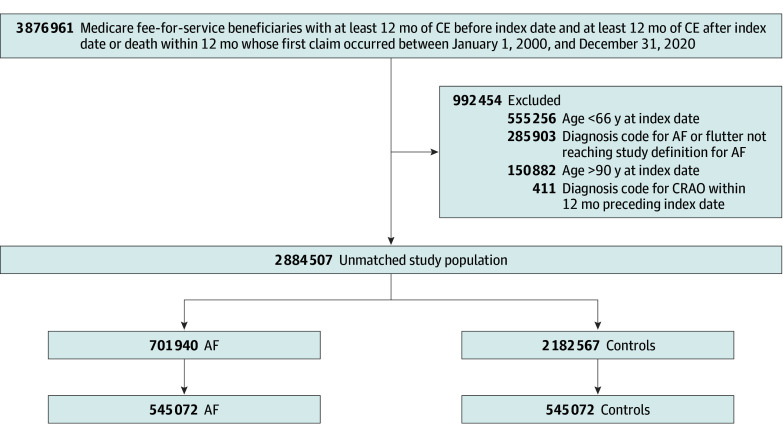
Flowchart of Patient Selection for Inclusion in the Study AF indicates atrial fibrillation; CE, continuous enrollment; and CRAO, central retinal artery occlusion.

A total of 1 090 144 patients (591 400 female [54.3%]; mean [SD] age, 76.92 [7.09] years) were included in the study; 545 072 patients had AF and 545 072 were matched controls. Figure 1 presents the flowchart of the study cohort derivation. Table 1 summarizes key clinical and demographic characteristics of the matched cohort. There were no significant differences across groups with respect to age, biological sex, race, ethnicity, and vascular risk factors. Baseline characteristics of the overlap weighted population are presented in eTable 1 in Supplement 1.

**Table 1.** Baseline Characteristics of the Study Cohort (Unweighted)^a^

Characteristic	Participants, No. (%)		ASD
	AF (n = 545 072)	Control (n = 545 072)	
Demographics			
Age, mean (SD), y	76.92 (7.09)	76.92 (7.09)	<0.001
Biological sex			
Female	291 348 (53.5)	300 052 (55.0)	0.007
Male	253 724 (46.5)	245 020 (45.0)	
Year of study entry			
2001-2005	188 038 (34.5)	188 038 (34.5)	<0.001
2006-2010	135 624 (24.9)	135 624 (24.9)	
2011-2015	120 040 (22.0)	120 040 (22.0)	
2016-2020	101 370 (18.6)	101 370 (18.6)	
Race and ethnicity			
Asian or Pacific Islander	5606 (1.0)	4987 (0.9)	0.014
Hispanic	6292 (1.2)	5992 (1.1)	
Non-Hispanic Black	33 232 (6.1)	33 319 (6.1)	
Non-Hispanic White	488 730 (89.7)	489 960 (89.9)	
Other^b^	11 212 (2.1)	10 814 (2.0)	
Medical history			
Hypertension	455 736 (83.6)	469 539 (86.1)	0.071
Hyperlipidemia	345 449 (63.4)	333 414 (61.2)	0.046
Coronary artery disease	264 615 (48.5)	270 923 (49.7)	0.023
Diabetes	188 928 (34.7)	193 079 (35.4)	0.016
Heart failure	172 634 (31.7)	153 680 (28.2)	0.076
Peripheral vascular disease	144 126 (26.4)	146 522 (26.9)	0.010
Cerebrovascular disease	118 380 (21.7)	120 604 (22.1)	0.010
Tobacco use	106 019 (19.5)	98 710 (18.1)	0.034
Chronic kidney disease	90 498 (16.6)	82 869 (15.2)	0.038

Abbreviations: AF, atrial fibrillation; ASD, absolute standardized difference.

^a^ Characteristics of the weighted cohort are presented in eTable 1 in Supplement 1.

^b^ Includes American Indian or Alaska Native, other, and unknown race.

### Follow-Up

The median (IQR) follow-up period was 45 (18-90) months. Reasons for loss to follow-up in the overlap weighting–derived AF group included the development of an end point (1333 participants), loss of coverage (252 051 participants), or death (291 688 participants). Reasons for loss to follow-up in the overlap weighting-derived control group included the development of an end point (1082 participants), loss of coverage or end of study period (277 323 participants), or death (257 863 participants).

### Primary End Point

**Figure 2.**
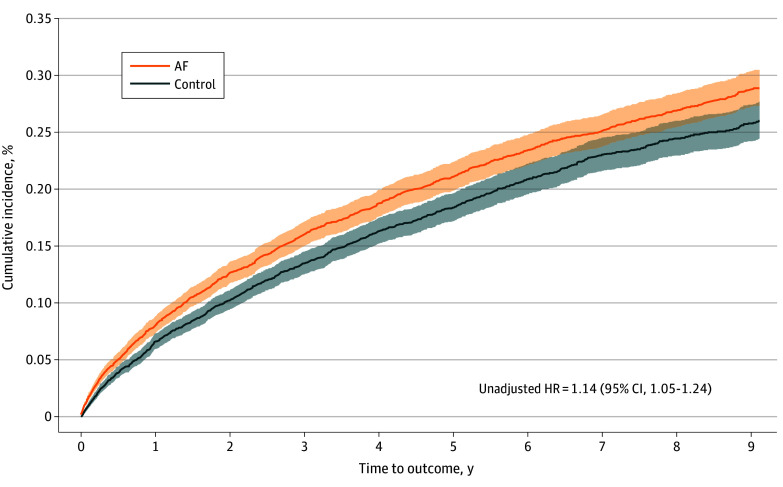
Cumulative Incidence of the Primary End Point According to Atrial Fibrillation (AF) Status Shaded areas denote 95% CIs. HR indicates hazard ratio.

Table 2 presents unadjusted and adjusted estimates of each end point in patients with and without AF. Of 545 072 patients with AF, 1333 (0.24%; rate, rate, 0.55 per 1000 person-years) experienced retinal stroke during follow up, compared with 1082 patients (0.20%; rate, 0.50 per 1000 person-years) without AF. In overlap weighted models, AF was associated with a 14% increase in the hazard of retinal stroke (cause-specific HR, 1.14 [95% CI, 1.02 to 1.28]; adjusted rate difference, 0.05 [95% CI, −0.01 to 0.11]). The cumulative incidence of retinal stroke is graphically depicted in Figure 2. The majority of claims for retinal stroke occurred in the office-based setting (2063 claims [85.42%]) with similar proportions in AF and control beneficiaries, followed by hospital outpatient clinics (181 claims [7.49%]), the ED (91 claims [3.77%]), inpatient facilities (47 claims [1.95%]), nursing facilities (0.80%), and skilled nursing facilities (0.50%) (exact numbers for nursing and skilled nursing facilities were redacted). The characteristics of beneficiaries with a claim for retinal stroke in the primary diagnostic position are summarized in eTable 2 in Supplement 1.

**Table 2.** Key Study End Points Among Patients With and Without AF

End point	Unadjusted				Adjusted rate/1000 person-years			Adjusted HR (95% CI)^a^
	AF (n = 545 072)		Control (n = 545 072)		AF	Control	Difference (95% CI)	
	No. of patients	Rate/1000 patient-years	No. of patients	Rate/1000 patient-years				
Primary end point								
Retinal stroke (primary diagnostic position)	1333	0.56	1082	0.50	0.55	0.50	0.05 (−0.01 to 0.11)	1.14 (1.02 to 1.28)
Secondary end points								
Retinal stroke (any diagnostic position)	2017	0.84	1689	0.78	0.84	0.78	0.06 (−0.02 to 0.13)	1.12 (1.02 to 1.23)
Any retinal artery occlusion^b^	9660	4.12	7665	3.61	4.14	3.60	0.54 (0.38 to 0.70)	1.21 (1.16 to 1.26)
Positive control end point								
Cerebral ischemic stroke	55 722	25.35	31 301	15.20	25.36	15.25	10.11 (9.72 to 10.49)	1.73 (1.69 to 1.76)
Negative control end points								
Central retinal vein occlusion	273	0.11	252	0.12	0.11	0.12	−0.0032 (−0.03 to 0.02)	1.00 (0.78 to 1.27)
Urinary tract infection	117 127	60.40	98 725	54.04	60.49	54.15	6.33 (5.65 to 7.02)	1.15 (1.13 to 1.16)
Cataract	29 651	13.27	24 128	11.82	13.26	11.82	1.45 (1.15 to 1.75)	1.15 (1.12 to 1.17)
Humeral fracture	6451	2.72	5333	2.49	2.72	2.49	0.23 (0.10 to 0.37)	1.12 (1.06 to 1.18)

Abbreviations: AF, atrial fibrillation; HR, hazard ratio.

^a^ Adjusted HRs are computed from a Cox model with AF (vs no AF) as the exposure. Overlap weighing was used to account for residual baseline imbalance after matching.

^b^ Any retinal artery occlusion includes central retinal artery occlusion, branch retinal artery occlusion, and other retinal artery occlusion.

### Secondary End Points

**Figure 3.**
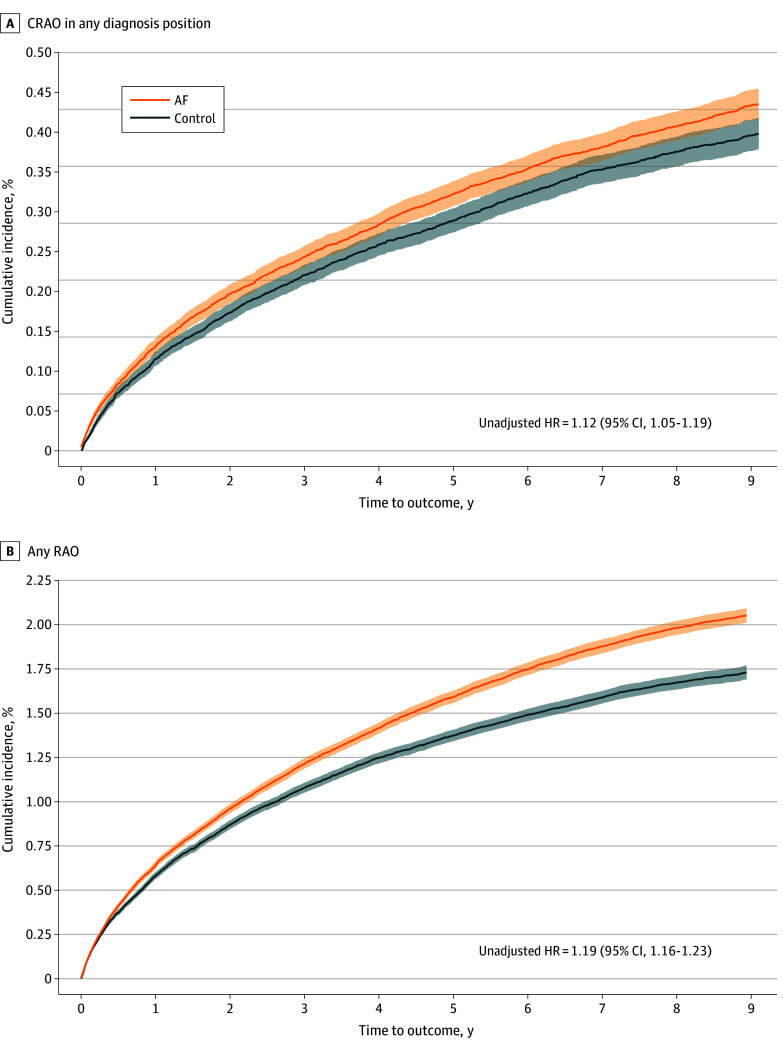
Cumulative Incidence of the Secondary End Points A, Graph shows incidence of central retinal artery occlusion (CRAO) in any diagnostic position. B, Graph shows incidence of any retinal artery occlusion (RAO; retinal stroke, branch retinal artery occlusion, and any retinal artery occlusion). Shaded areas denote 95% CIs. AF indicates atrial fibrillation; and HR, hazard ratio.

AF was positively associated with retinal stroke when considered in any diagnostic position (aHR, 1.12 [95% CI, 1.02 to 1.23]; adjusted rate difference, 0.06 [95% CI, −0.02 to 0.13]). In addition, AF was associated with a 21% increased hazard of any retinal artery occlusion (aHR, 1.21 [95% CI, 1.16 to 1.26]; adjusted rate difference, 0.54 [95% CI, 0.38 to 0.70]). The cumulative incidence of each secondary end point is graphically depicted in Figure 3.

### Positive and Negative Control End Points

For the positive control end point, there was an association between AF and cerebral ischemic stroke, which occurred in 55 722 patients with AF (10.22%) vs 31 301 control patients (5.71%) (aHR, 1.73 [95% CI, 1.69 to 1.76]; adjusted rate difference, 10.11 [95% CI, 9.72 to 10.49]). With respect to negative control end points, there was no observed association between AF and central retinal vein occlusion (aHR, 1.00 [95% CI, 0.78 to 1.27]; adjusted rate difference, 0.01 [95% CI, −0.03 to 0.02]). By contrast, there was an association between AF and UTI (aHR, 1.15 [95% CI, 1.13 to 1.16]; adjusted rate difference, 6.33 [95% CI, 5.65 to 7.02]), cataract (aHR, 1.15 [95% CI, 1.12 to 1.17]; adjusted rate difference, 1.45 [95% CI, 1.15 to 1.75]), and humeral fracture (aHR, 1.12 [95% CI, 1.06 to 1.18]; adjusted rate difference, 0.23 [95% CI, 0.10 to 0.37]).

### Falsification Exposure

A falsification exposure analysis was conducted. Among 627 898 patients with an acute UTI, 1198 (0.19%) developed retinal stroke compared with 1179 of 627 898 (0.19%) matched UTI-free control patients (aHR, 0.88 [95% CI, 0.79 to 0.99]; adjusted rate difference, −0.07 [95% CI, −0.11 to −0.02]).

### Sensitivity Analysis

We also conducted a sensitivity analysis. The results of this analysis were unchanged when censoring was not conducted at 85% loss to follow-up of the entire cohort (eTable 3 in Supplement 1).

## Discussion

In this population-based cohort study of Medicare beneficiaries aged 66 years and older, AF was independently associated with the hazard of retinal stroke. Key methodological attributes of this study include the inclusion of a diverse population of Medicare beneficiaries, a prespecified statistical analysis plan, and the inclusion of claims from multiple venues of care (office care, hospital-based outpatient clinic, ED, inpatient setting, and short-term and long-term nursing facilities). This study occurs in the context of several prior studies. A population-based cohort study^15^ using data Taiwan’s National Health Insurance program examined the hazard of retinal stroke in 9756 patients with AF compared with 38 872 AF-free controls. In that study, an analysis adjusted for age and biological sex established that AF was associated with retinal stroke (aHR, 8.32 [95% CI, 3.70 to 18.32]) but did not account for potentially important domains of confounding, including the presence of other vascular risk factors (eg, hypertension, hyperlipidemia, diabetes, and smoking). Our previous study^16^ included 39.8 million people who were discharged from nonfederal hospital facilities in New York, California, and Florida. Of this cohort, 2.7 million had hospital-documented AF and 37.1 million did not have hospital-documented AF. Although there was an unadjusted association between AF and retinal stroke (HR, 2.55 [95% CI, 2.15 to 3.03]) there was an inverse association between AF and retinal stroke after adjustment for age, biological sex, state, and a suite of vascular comorbidities. The low observed number of retinal stroke events (compared with prior epidemiologic studies^29^) and secular trends in coding behavior supports the idea of underascertainment of this end point in claims data.

This study has several key methodological attributes. First, it is population-based, incorporating a random sample of Medicare beneficiaries from across the US. Second, the incorporation of multiple domains of care greatly increases the ascertainment of retinal stroke claims. In the present study, 92.91% of retinal stroke claims were from independent offices or hospital outpatient clinics. Third, a multistage approach to covariate adjustment was adopted, including propensity score matching followed by overlap weighting, which yielded excellent covariate balance. The association of AF with ischemic stroke is lower in the present study (aHR, 1.73 [95% CI, 1.69 to 1.76]) than in prior studies (eg, studies based on the Framingham cohort suggesting that vascular risk was accounted for to a greater extent than in prior studies).^2^ In this study, AF was associated with 3 of 4 falsification end points: UTI, cataract, and humeral fracture. Although an increased hazard of UTI or humeral fracture may be mediated by an increased risk of ischemic stroke, syncope, hospitalization in general, or immobility, this conclusion cannot be made on the basis of the results presented. The increased hazard of cataract diagnosis raises the specter of increased contact with overall ophthalmological care as a potential unmeasured difference between the AF and control groups.

### Strengths and Limitations

There are several limitations inherent in this study. First, this study was conducted in a population of Medicare fee-for-service beneficiaries aged 66 years or older, and is it not clear that the study findings generalize outside of this population. Second, the possibility of residual, unmeasured confounding remains, which is of particular note given the small magnitude of the observed association between AF and retinal stroke. Third, although the diagnosis code for retinal stroke has been used in multiple prior studies^15,30,31,32,33,34^ and validated in the setting of inpatient claims,^16^ it remains to be validated for use in outpatient settings and other venues of care (including short-term and long-term nursing facilities). This raises the concern that other disorders with partially overlapping presenting features, such as nonarteritic ischemic optic neuropathy, may have been inadvertently miscoded as retinal stroke. However, the presence of only a single diagnosis code for retinal stroke and straightforward *ICD-9-CM* to *ICD-10-CM* conversion diminishes this concern. Fourth, censoring was assumed to be noninformative. Fifth, given the high prevalence of undiagnosed AF, it is possible that the matched cohort of control beneficiaries may have included beneficiaries with undiagnosed AF, a conservative bias in the study. Sixth, the interplay between AF, anticoagulation, and retinal stroke remains to be explored and may shed important light on this question. Seventh, the data presented cannot disentangle a temporal association between AF and retinal stroke or an association between overall AF burden and retinal stroke, 2 factors known to be associated with cerebral ischemic stroke.^35,36^

## Conclusions

In this cohort study of Medicare beneficiaries aged 66 years and older, AF was independently associated with retinal stroke. A contribution from residual, unmeasured confounding could not be excluded.

## Notes

Supplement 1. **eTable 1.** Baseline Characteristics of the Study Cohort (Weighted) **eTable 2.** Clinical Characteristics of Beneficiaries with Retinal Stroke **eTable 3.** Key Study End Points in Patient With and Without Atrial Fibrillation (Sensitivity Analysis Without Censoring at 85% Loss to Follow-Up)

Supplement 2.
Data Sharing Statement
