# Supplementary material for: Atrial Fibrillation and Retinal Stroke
Source: JAMA Netw Open. 2025 Jan 9;8(1):e2453819. doi: 10.1001/jamanetworkopen.2024.53819 (PMC11718556; doi:10.1001/jamanetworkopen.2024.53819)
Supplement: Supplement 1. — eTable 1. Baseline Characteristics of the Study Cohort (Weighted) eTable 2. Clinical Characteristics of Beneficiaries with Retinal Stroke eTable 3. Key Study End Points in Patient With and Without Atrial Fibrillation (Sensitivity Analysis Without Censoring at 85% Loss to Follow-Up) [file jamanetwopen-e2453819-s001.pdf]

Supplemental Online Content

Lusk JB, Nalawade V, Wilson LE, et al. Atrial fibrillation and retinal stroke. *JAMA Netw Open*. 2025;8(1):e2453819. doi:10.1001/jamanetworkopen.2024.53819

eTable 1. Baseline Characteristics of the Study Cohort (Weighted)

eTable 2. Clinical Characteristics of Beneficiaries with Retinal Stroke

eTable 3. Key Study End Points in Patient With and Without Atrial Fibrillation (sensitivity analysis without censoring at 85% loss to follow-up)

This supplemental material has been provided by the authors to give readers additional information about their work.

© 2025 Lusk JB et al. *JAMA Network Open*.

eTable 1. Baseline Characteristics of the Study Cohort (Weighted).

Characteristic	AF (n = 271,349.66)	Control (n = 271,349.66)	ASD
**Demographics**			
Age (mean, SD)	76.91 (7.08)	76.91 (7.10)	<0.001
Biological Sex			<0.001
Women (number, %)	147,390.6 (54.3)	147,390.6 (54.3)	
Men (number, %)	123,959.06 (45.7)	123,959.06 (45.7)	
Year of study entry			0.013
2001-2005	92,977.3 (34.3)	94,269.3 (34.7)	
2006-2010	68,087.7 (25.1)	66,929.3 (24.7)	
2011-2015	60,050.6 (22.1)	59,417.4 (21.9)	
2016-2020	50,234.1 (18.5)	50,733.6 (18.7)	
Race/Ethnicity			<0.001
Asian/Pacific Islander	2,634.4 (1.0)	2,634.4 (1.0)	
Hispanic	3,066.6 (1.1)	3,066.6 (1.1)	
Non-Hispanic Black	16,609.2 (6.1)	16,609.2 (6.1)	
Non-Hispanic White	243,556.1 (89.8)	243,556.1 (89.8)	
Other[a]	5,483.4 (2.0)	5,483.4 (2.0)	
**Past Medical History**			
Hypertension	230,613.9 (85.0)	230,613.9 (85.0)	<0.001
Hyperlipidemia	169,099.3 (62.3)	169,099.3 (62.3)	<0.001
Coronary artery disease	80,869.1 (29.8)	80,869.1 (29.8)	<0.001
Diabetes mellitus	95,161.7 (35.1)	95,161.7 (35.1)	<0.001
Heart failure	80,869.1 (29.8)	80,869.1 (29.8)	<0.001
Peripheral vascular disease	72,339.7 (26.7)	72,339.7 (26.7)	<0.001
Cerebrovascular disease	59,513.4 (21.9)	59,513.4 (21.9)	<0.001
Tobacco use	50,832.5 (18.7)	50,832.5 (18.7)	<0.001
Chronic kidney disease	43,085.4 (15.9)	43,085.4 (15.9)	<0.001

Abbreviations: AF, atrial fibrillation; ASD; absolute standardized difference; SD, standard deviation.

[a]Includes North American Native, other, and unknown race.

© 2025 Lusk JB et al. *JAMA Network Open*.

eTable 2. Clinical Characteristics of Beneficiaries with Retinal Stroke.

Characteristic	Total (n = 2,415)	AF (n = 1,333)	Control (n = 1,082)
**Demographics**			
Age (mean, SD)	77.34 (6.65)	77.03 (6.55)	77.72 (6.75)
Biological Sex			
Women (number, %)	1250 (51.8)	661 (49.6)	589 (54.4)
Men (number, %)	1165 (48.2)	672 (50.4)	493 (45.6)
Year of study entry			
2001-2005	962 (39.8)	565 (42.4)	397 (36.7)
2006-2010	641 (26.5)	335 (25.1)	306 (28.3)
2011-2015	560 (23.2)	295 (22.1)	265 (24.5)
2016-2020	252 (10.4)	138 (10.4)	114 (10.5)
Race/Ethnicity			
Asian/Pacific Islander	22 (0.9)	R[a]	R[a]
Hispanic	18 (0.7)	R[a]	R[a]
Non-Hispanic Black	116 (4.8)	66 (5.0)	50 (4.6)
Non-Hispanic White	2214 (91.7)	1223 (91.7)	991 (91.6)
Other[b]	45 (1.9)	24 (1.8)	R[a]
**Location**			
Office location	2,063 (85.42)	1,138 (85.37)	925 (85.49)
Outpatient claim	181 (7.49)	95 (7.13)	86 (7.95)
ED claim	91 (3.77)	52 (3.90)	39 (3.60)
Inpatient claim	47 (1.95)	33 (2.48)	14 (1.29)
NF location	R[a]	R[a]	R[a]
SNF claim	R[a]	R[a]	R[a]
Home Location	R[a]	R[a]	R[a]
Custodial care facility	R[a]	R[a]	R[a]

Abbreviations: AF, atrial fibrillation; R, redacted; SD, standard deviation.

a. Redacted in keeping with the Centers for Medicare and Medicaid Services Cell Suppression Policy.

b. Includes North American Native, other, and unknown race.

© 2025 Lusk JB et al. *JAMA Network Open*.

eTable 3. Key Study End Points in Patient With and Without Atrial Fibrillation (sensitivity analysis without censoring at 85% loss to follow-up)

	Unadjusted				Adjusted			
	AF (n = 545,072)		Control (n = 545,072)		Rate/1,000 person- years			
	No.	Rate/1,000 person-years	No.	Rate/1,000 person-years	AF	Control	Hazard Ratio (95% CI)[a]	Rate Difference (95% CI)
**Primary End Point**								
CRAO (Primary Diagnostic Position)	1,468	0.54	1,175	0.50	0.54	0.50	1.13 (1.01 – 1.26)	0.04 (-0.01 – 0.10)
**Secondary End Point**								
CRAO (Any Diagnostic Position)	2,216	0.81	1,812	0.76	0.81	0.76	1.12 (1.02 – 1.22)	0.05 (-0.02 – 0.12)
Any RAO*	10,229	3.82	7,929	3.39	3.84	3.38	1.22 (1.17 – 1.27)	0.46 (0.31 – 0.61)
**Positive Control End Point**								
Cerebral Ischemic Stroke	59,704	23.61	32,847	14.35	23.61	14.40	1.73 (1.70 – 1.77)	9.21 (8.86 – 9.56)
**Negative Control End Points**								
CRVO	314	0.11	276	0.12	0.11	0.12	1.01 (0.80 – 1.27)	-0.0015 (-0.03 – 0.02)
UTI	132,492	58.62	108,229	52.68	58.72	52.76	1.15 (1.14 – 1.16)	5.96 (5.32 – 6.59)
Cataract	32,633	12.72	25,787	11.40	12.71	11.40	1.15 (1.12 – 1.18)	1.31 (1.03 – 1.58)
Humeral Fracture	6,974	2.58	5,664	2.40	2.58	2.40	1.12 (1.06 – 1.17)	0.18 (0.06 – 0.30)

Abbreviations: AF, atrial fibrillation; CI, confidence interval; UTI, urinary tract infection.

[a]Adjusted hazard ratios are computed from a Cox model with AF (vs. no AF) as the exposure. Overlap weighing was used to account for residual baseline imbalance after matching.

© 2025 Lusk JB et al. *JAMA Network Open*.
