# Supplementary material for: Atrial Fibrillation and Retinal Stroke
Source: JAMA Netw Open. 2025 Jan 9;8(1):e2453819. doi: 10.1001/jamanetworkopen.2024.53819 (PMC11718556; doi:10.1001/jamanetworkopen.2024.53819)
Supplement: Supplement 2. — Data Sharing Statement [file jamanetwopen-e2453819-s002.pdf]

Data Sharing Statement

Lusk. Atrial Fibrillation and Retinal Stroke. *JAMA Netw Open*. Published January 09, 2025.
doi:10.1001/jamanetworkopen.2024.53819

Data
Data available: No

Additional Information
Explanation for why data not available: This study was conducted under the auspices of a data usage agreement (DUA) between Duke University and the Centers for Medicare and Medicaid Services (CMS; DUA# RSCH-2023-59144). DUAs require an application process in order for other researchers to access the data.
